# Television and computer use and dementia risk in older adults with limited leisure or social activities: A prospective cohort study

**DOI:** 10.1002/alz.71259

**Published:** 2026-03-08

**Authors:** Jiaying Li, Hongliang Xue, Yue Leng, Quincy M. Samus, Milap Nowrangi, Sarah L. Szanton, Qian‐Li Xue, Juxnin Li

**Affiliations:** ^1^ The Nethersole School of Nursing Faculty of Medicine The Chinese University of Hong Kong, Shatin, New Territories Hong Kong SAR China; ^2^ Department of Nutrition School of Public Health Guangzhou Medical University Guangzhou Guangdong China; ^3^ Department of Psychiatry and Behavioral Sciences University of California San Francisco California US; ^4^ School of Nursing Johns Hopkins University Baltimore Maryland US; ^5^ Department of Psychiatry and Behavioral Sciences Johns Hopkins University School of Medicine Baltimore Maryland US; ^6^ Department of Epidemiology Johns Hopkins Bloomberg School of Public Health Baltimore Maryland US

**Keywords:** computer use, dementia risk, screen‐time thresholds, social inactivity, television viewing

## Abstract

**INTRODUCTION:**

Associations between television/computer use and dementia in socially inactive older adults remain unclear, and optimal limits are unknown.

**METHODS:**

We followed 89,671 dementia‐free, socially inactive adults aged ≥55 from UK Biobank for a mean of 12.2 years. Adjusted Cox models assessed associations with incident all‐cause dementia and subtypes.

**RESULTS:**

Computer use ≤2.4 h/day was associated with lower all‐cause dementia risk (hazard ratio [HR] 0.88; 95% confidence interval [CI] 0.82–0.94), whereas higher use increased risk (HR 1.19, 95% CI 1.05–1.34); patterns were similar for Alzheimer's and vascular dementia. Television viewing showed no association below 2.06 h/day but higher risk thereafter (HR 1.17; 95% CI 1.03–1.32), with a roughly linear increase for vascular dementia. Heavy computer use in apolipoprotein E (*APOE*) ‐ε4 homozygotes and higher television viewing in adults < 65 were more harmful.

**DISCUSSION:**

In socially inactive older adults, moderate computer use may be protective, whereas higher computer use and television viewing are linked to increased dementia risk.

## Highlights

- Among socially inactive older adults, computer use showed a J‐shaped association with all‐cause dementia: up to about 2.4 h/day was associated with lower risk, whereas higher use was associated with increased risk.
- The J‐shaped pattern extended to Alzheimer's disease with an inflection near 2.4 h/day and to vascular dementia with an inflection near 2.2 h/day, while no association was observed for frontotemporal dementia.
- Television viewing below about 2.1 h/day was not associated with higher all‐cause dementia risk, but risk increased at higher viewing times.
- Television viewing showed a roughly linear increase in vascular dementia risk and an apparent reduction in Alzheimer's disease risk that is likely spurious and should be interpreted with caution.
- Risk varied by subgroup: heavy computer use was particularly harmful among apolipoprotein E (*APOE*) ‐ε4 homozygotes, and higher television time was more detrimental in adults younger than 65 years.

## 1 INTRODUCTION

Dementia poses an escalating global challenge—57 million cases, 2 million deaths and 36.3 million disability‐adjusted life years in 2021, projected to reach 153 million cases by 2050.^1^, ^2^ With no disease‐modifying therapies, up to 40% of dementia risk is attributable to modifiable behaviors, making prevention via lifestyle change imperative.^3^ Concurrently, digital adoption among older adults has surged, from around 10% to over 70% internet use in Switzerland and from 12% to two‐thirds in the United States over two decades,^4^ yet television (TV) viewing still occupies 25%–30% of waking hours and up to half of leisure time.^5^ The coronavirus disease 2019 (COVID‐19) pandemic's acceleration of digital uptake^6^ underscores the urgency of distinguishing passive and interactive screen modalities to inform healthy‐ageing strategies.

Screen‐based leisure activities—mainly TV viewing and computer use—are widespread yet modifiable in older adults. Passive TV watching has been associated with faster cognitive decline and higher dementia risk,^7^ whereas interactive computer activities (e.g., reading, gaming) are associated with lower risk independent of physical activity.^8^, ^9^, ^10^ This contrast suggests that cognitive engagement, rather than inactivity alone, may be more salient for neurocognitive outcomes. While earlier studies, including those utilizing UK Biobank data,^7^, ^8^, ^11^ often assumed a linear relationship, recent analyses of this same cohort by Zhuang et al., Wu et al., and Xu et al. have challenged this paradigm,^12^, ^13^, ^14^ identifying modality‐specific, non‐linear dose‐response curves with apparent inflection points. However, these pivotal studies focused on the general population (aged 37–73). Crucially, they generally did not adjust for key genetic confounders such as apolipoprotein E (*APOE*) ‐ε4 or examine specific dementia subtypes,^12^, ^13^, ^14^ limiting the applicability of their findings to vulnerable subgroups.

Consequently, it remains unknown whether thresholds derived from general cohorts ^12^, ^13^, ^14^ apply to socially inactive older adults, defined by a lack of engagement in social interactions or group activities,^15^ or to particular dementia subtypes such as Alzheimer's disease (AD), vascular dementia (VD), and frontotemporal dementia (FTD). In general cohorts, moderate daily computer use often reflects broader cognitive and social stimulation and is linked to greater cognitive reserve,^8^, ^12^ and a 40%–50% lower dementia risk.^16^ By contrast, socially inactive seniors—those reporting no leisure or social activities—face approximately a 30% higher dementia risk from low social contact alone,^17^, ^18^ suggesting that the same “dose” of screen time may be insufficient, or even detrimental, without other engagement. Age‐related declines in neuroplasticity could further shift these optimal thresholds in this subgroup. Yet existing studies rely on linear or coarse categories, often omit key confounders such as *APOE*‐ε4, and seldom examine subtype‐ or high‐risk subgroup effects. Addressing these gaps requires flexible spline modelling, comprehensive multivariable adjustment, and stratified interaction analyses in this specific vulnerable population.

Therefore, in a prospective cohort of adults aged ≥55 years with no leisure or social activities, we aim to: (1) examine TV and computer use in relation to incident all‐cause and subtype dementia, (2) employ restricted cubic splines to map non‐linear dose–response curves and inflection points, and (3) perform stratified interaction analyses to identify high‐risk subgroups. These findings are expected to characterize modality‐specific associations and inform risk stratification in socially inactive seniors.

## 2 METHODS

### 2.1 Study population and design

We analyzed data from UK Biobank, a prospective cohort of > 500 000 adults aged 37–73 years recruited at 22 centers across England, Scotland, and Wales from April 2007 to December 2010. At baseline, participants completed touchscreen questionnaires on sociodemographics, lifestyle, and medical history, and underwent anthropometric measurements by trained staff. Additionally, at baseline, blood samples were collected for central assays, including non‐fasting triglycerides, total cholesterol, glucose, and high density lipoprotein cholesterol (HDL‐C).^19^ The UK Biobank study protocol received approval from the North West Multi‐center Research Ethics Committee (11/NW/0382), the study was conducted under Application Number 755949. More details concerning the UK Biobank can be obtained online.

We excluded participants aged < 55 years (*n* = 194 140). Given the mean follow‐up of ∼12 years, this cutoff ensures the cohort reaches the high‐risk age for late‐onset dementia (≥65 years) while targeting the critical intervention window around retirement, a pivotal and actionable period for lifestyle restructuring to preserve cognitive reserve. Then, to define the socially inactive group, we assessed weekly participation in group activities using the baseline touchscreen questionnaire. Participants were asked, “Which of the following do you attend once a week or more often?” with options including: (1) sports club or gym, (2) pub or social club, (3) religious group, (4) adult education class, or (5) other group activity. We excluded all participants reporting engagement in any of these categories (*n* = 216 937). The final analytical sample consisted of those selecting “None of the above,” yielding 89 671 participants after further removing those with missing reports of TV or computer use (*n* = 1 631) and prevalent dementia (*n* = 33) (Figure S1).

### 2.2 Exposures

Exposures were self‐reported at baseline via touchscreen: “In a typical day, how many hours do you spend watching TV?” and “…using the computer? (Excluding work).” If estimates varied across days, participants provided a 4‐week average. Responses < 0 or > 24 h were rejected; responses > 6 h prompted confirmation. “Less than an hour” was coded as 0.5 h; “do not know” or “prefer not to answer” were set to missing. Both exposures were treated as continuous variables.

### 2.3 Outcomes

Incident dementia—both the event (all‐cause and subtype: AD, VD, FTD, and other) and time to first diagnosis—was ascertained from linked national hospital inpatient datasets (Hospital Episode Statistics for England; Scottish Morbidity Record for Scotland; Patient Episode Database for Wales) and death registries (National Health Service Digital for England & Wales; Information and Statistics Division for Scotland) using International Classification of Diseases‐9/10 codes (Table S1).

### 2.4 Covariates

Covariates included: (1) demographics: sex, baseline age (continuous), education (<  high school, high school, ≥ college), ethnicity (white vs non‐white), Townsend Deprivation Index (≤ –2 [less deprived], –2 to 2 [normal], > 2 [most deprived]);^20^ (2) lifestyle: physical activity (≥ 150 min/week moderate or ≥ 75 min/week vigorous),^21^ smoking status (never, former, current), alcohol intake (never, former, current), family/friend visit frequency (>  1/month vs ≤ 1/month); (3) health conditions: obesity (body mass index ≥ 30 kg/m^2^), hypertension (systolic blood pressure  >  130 mm Hg or diastolic blood pressure  >  85 mm Hg or self‐reported), diabetes (fasting glucose  >  5.6 mmol/L or self‐reported), high triglycerides (>  1.7 mmol/L), low HDL‐C (<  1.0 mmol/L in men, <  1.3 mmol/L in women), hearing impairment (self‐reported yes vs no); (4) genetics: *APOE* genotype was categorized into four groups—ε3 homozygotes (ε3/ε3) as the reference, ε2‐only carriers (ε2/ε2 or ε2/ε3), single ε4 carriers (ε2/ε4 or ε3/ε4), and double ε4 homozygotes (ε4/ε4)^22^.

**RESEARCH IN CONTEXT**

1. **Systematic review**: Prior research has established that passive television (TV) viewing is generally linked to cognitive decline, while interactive computer use appears protective. However, most studies assumed a linear dose–response relationship. While a few recent studies have begun to reveal nonlinear, dose‐dependent relationships for screen time, these findings had not explored this high‐risk population of socially inactive older adults nor tested associations with dementia subtypes. Critical gaps remained in understanding how these nonlinear risks differed across dementia subtypes (such as Alzheimer's disease and vascular dementia) and whether they were modified by key genetic factors like the apolipoprotein E (*APOE*) ‐ε4 genotype.
2. **Interpretation**: This study provides granular evidence by analyzing a large cohort of 89,671 socially inactive older adults over 12.2 years. We extend prior work by demonstrating a nonlinear, J‐shaped relationship for computer use specifically within this vulnerable group: moderate use (up to 2.4 h/day) was associated with a lower risk, while higher use was linked to an increased risk. In contrast, TV viewing was associated with a higher risk beyond 2 h/day. We also identified specific vulnerabilities, showing that high computer use was particularly detrimental for *APOE*‐ε4 homozygotes, while high TV viewing posed a greater risk for adults younger than 65 years.
3. **Future directions**: Our findings suggest that for socially inactive seniors, the type and dose of screen time are critical. The identified thresholds can help clinicians stratify risk and provide more nuanced advice, moving away from generic guidance to recommend moderate, cognitively engaging screen time while discouraging prolonged passive viewing. While these observational results are hypothesis‐generating, they strongly suggest that screen‐use behaviors are a candidate target for intervention. The next step is to conduct randomized or pragmatic trials to determine whether modifying screen habits, such as promoting interactive tasks within optimal limits, causally reduces dementia risk in this population.

### 2.5 Statistical analyses

We conducted a five‐step analysis to (1) characterize our cohort, (2) quantify exposure–dementia associations, (3) assess non‐linearity and identify inflection points, (4) evaluate interaction effects between computer use and TV viewing, (5) uncover high‐risk subgroups, and (6) test robustness via sensitivity analyses. First, we described participant characteristics by quartiles of TV and computer use (continuous as mean ± standard deviation; categorical as *n* [%]) and assessed covariate balance via standardized mean differences (SMD; |SMD| < 0.10 negligible; > 0.20 moderate) alongside *χ* ^2^ tests or analysis of variance (ANOVA).^23^ Second, we fitted three nested Cox proportional‐hazards models for each exposure–outcome pair: Model 1 adjusted for age, sex, and *APOE*‐ε4; Model 2 was additionally adjusted for education, social visits, ethnicity, Townsend index, physical activity, smoking, and alcohol; Model 3 further for hearing impairment, obesity, diabetes, hypertension, triglycerides, and total cholesterol. Variance inflation factors (VIFs) < 5 indicated no collinearity.^24^ Third, to assess non‐linearity, we replaced each continuous exposure in Model 3 with a restricted cubic spline (four knots), tested overall, linear, and non‐linear components by ANOVA, and plotted adjusted hazard‐ratio curves with 95% confidence intervals (CIs). For models showing non‐linearity, we identified inflection points via grid search, fitted piecewise Cox models below and above each threshold, and compared them with standard linear models using likelihood‐ratio tests. Fourth, we examined the interplay between the two screen behaviors by testing the multiplicative interaction between TV viewing and computer use and estimating hazard ratios for their combined usage categories. Fifth, we dichotomized exposures at spline‐derived thresholds and, in fully adjusted Cox models, estimated high‐versus‐low hazard ratios within strata of pre‐specified covariates (sex; age < 65 vs ≥ 65; *APOE*‐ε4 status; education; social visits; ethnicity; deprivation; physical activity; smoking; alcohol; hearing; obesity; diabetes; hypertension; lipid levels) and tested effect modification via likelihood‐ratio interaction tests. Finally, we confirmed robustness and explored potential pathways with five sensitivity analyses: (1) imputing missing covariates using multiple imputation by chained equations (MICE; five imputed datasets, pooled by Rubin's rules)—missing rates and imputation details are shown in Table S2; (2) applying a 2‐year landmark analysis to mitigate reverse causality; (3) fitting Fine–Gray subdistribution‐hazard models to account for competing mortality; (4) additionally adjusting for sleep duration (normal [7–9 h] vs. non‐normal) and depressive symptoms (dichotomized PHQ‐2 score) to evaluate whether associations were independent of these potential confounders and mediators; and (5) validating the specificity of our findings by repeating the primary models for all‐cause dementia in the excluded subgroup of socially active participants to determine if social engagement modifies the observed risk trajectories. Analyses were conducted in R v4.1.1, and two‐sided *p *< 0.05 was considered significant.

## 3 RESULTS

### 3.1 Characteristics of study participants

Among 89,671 adults (mean age 61.8 ± 4.1 years; 47.0% male), mean leisure computer use was 1.00 ± 1.38 h/day and TV viewing 3.23 ± 1.80 h/day. Most covariates differed across computer and TV quartiles (all *p* < 0.05), except *APOE* status across TV viewing. SMDs indicated moderate imbalance (|SMD| > 0.20) for education and sex across computer‐use quartiles, and for education, obesity, and total cholesterol across TV viewing quartiles (Table 1).

**TABLE 1:** Baseline characteristics by quartiles of daily computer use and television viewing.

Characteristics	Computer use (h/day)						Television viewing (h/day)					
	Q1 (0–0)	Q2 (0–0.5)	Q3 (0.5–1)	Q4 (1–20)	*p*‐value	SMD	Q1 (0–2)	Q2 (2–3)	Q3 (3–4)	Q4 (4–20)	*p*‐value	SMD
*n*	22000	21999	21999	21999			22146	22146	22145	22145		
Age (years), mean (SD)	62.40 (4.13)	61.68 (4.07)	61.29 (4.00)	61.62 (4.02)	<0.001	0.14	61.09 (4.02)	61.48 (4.02)	61.92 (4.09)	62.47 (4.05)	<0.001	0.19
*APOE* genotype, *n* (%)					0.042	0.02					0.882	0.01
ε3 homozygotes	12503 (56.8)	12554 (57.1)	12817 (58.3)	12590 (57.2)			12695 (57.3)	12578 (56.8)	12576 (56.8)	12643 (57.1)		
ε2 carriers without ε4	2817 (12.8)	2810 (12.8)	2765 (12.6)	2864 (13.0)			2788 (12.6)	2825 (12.8)	2846 (12.9)	2802 (12.7)		
Single ε4 carrier	5574 (25.3)	5640 (25.6)	5482 (24.9)	5526 (25.1)			5581 (25.2)	5595 (25.3)	5583 (25.2)	5496 (24.8)		
ε4 homozygotes	537 (2.4)	511 (2.3)	478 (2.2)	448 (2.0)			493 (2.2)	502 (2.3)	512 (2.3)	466 (2.1)		
Missing	569 (2.6)	484 (2.2)	457 (2.1)	571 (2.6)			589 (2.7)	646 (2.9)	628 (2.8)	738 (3.3)		
Education level, *n* (%)					<0.001	0.46					<0.001	0.47
Less than high school	10503 (47.7)	7084 (32.2)	4073 (18.5)	4117 (18.7)			3578 (16.2)	5328 (24.1)	7140 (32.2)	9766 (44.1)		
High school or equivalent	2193 (10.0)	4365 (19.8)	6747 (30.7)	7589 (34.5)			8899 (40.2)	5743 (25.9)	3864 (17.4)	2377 (10.7)		
College or above	8852 (40.2)	10263 (46.7)	10983 (49.9)	10070 (45.8)			9279 (41.9)	10666 (48.2)	10662 (48.1)	9553 (43.1)		
Missing	452 (2.1)	287 (1.3)	196 (0.9)	223 (1.0)			390 (1.8)	409 (1.8)	479 (2.2)	449 (2.0)		
Family and friend visit frequency, *n* (%)					<0.001	0.05					<0.001	0.05
Infrequent visits	2291 (10.4)	2093 (9.5)	1957 (8.9)	2545 (11.6)			2458 (11.1)	2108 (9.5)	1931 (8.7)	2389 (10.8)		
Frequent visits	19499 (88.6)	19778 (89.9)	19960 (90.7)	19325 (87.8)			19410 (87.6)	19792 (89.4)	19937 (90.0)	19450 (87.8)		
Missing	210 (1.0)	128 (0.6)	82 (0.4)	129 (0.6)			278 (1.3)	246 (1.1)	277 (1.3)	306 (1.4)		
Ethnicity, n (%)					<0.001	0.05					<0.001	0.03
White	21028 (95.6)	21214 (96.4)	21317 (96.9)	20971 (95.3)			21051 (95.1)	21318 (96.3)	21373 (96.5)	21297 (96.2)		
Other	909 (4.1)	726 (3.3)	601 (2.7)	914 (4.2)			973 (4.4)	762 (3.4)	709 (3.2)	783 (3.5)		
Missing	63 (0.3)	59 (0.3)	81 (0.4)	114 (0.5)			122 (0.6)	66 (0.3)	63 (0.3)	65 (0.3)		
Townsend Deprivation Index, *n* (%)					<0.001	0.16					<0.001	0.14
≤ −2	9857 (44.8)	11369 (51.7)	12477 (56.7)	11040 (50.2)			11460 (51.7)	12140 (54.8)	11573 (52.3)	9829 (44.4)		
> −2 to ≤ 2	7201 (32.7)	6967 (31.7)	6810 (31.0)	7146 (32.5)			7107 (32.1)	6978 (31.5)	7088 (32.0)	7117 (32.1)		
> 2	4916 (22.3)	3641 (16.6)	2691 (12.2)	3782 (17.2)			3550 (16.0)	3000 (13.5)	3466 (15.7)	5174 (23.4)		
Missing	26 (0.1)	22 (0.1)	21 (0.1)	31 (0.1)			29 (0.1)	28 (0.1)	18 (0.1)	25 (0.1)		
Sex, *n* (%)					<0.001	0.27					<0.001	0.08
Female	13722 (62.4)	13308 (60.5)	10881 (49.5)	8634 (39.2)			10796 (48.7)	11480 (51.8)	12195 (55.1)	12293 (55.5)		
Male	8278 (37.6)	8690 (39.5)	11118 (50.5)	13365 (60.8)			11350 (51.3)	10665 (48.2)	9950 (44.9)	9852 (44.5)		
Missing	0 (0.0)	1 (0.0)	0 (0.0)	0 (0.0)			0 (0.0)	1 (0.0)	0 (0.0)	0 (0.0)		
Physical activity, *n* (%)					<0.001	0.09					<0.001	0.08
Low activity	9562 (43.5)	10227 (46.5)	10657 (48.4)	11631 (52.9)			10161 (45.9)	10337 (46.7)	10339 (46.7)	11465 (51.8)		
High activity	11198 (50.9)	10874 (49.4)	10908 (49.6)	9837 (44.7)			11470 (51.8)	11191 (50.5)	11019 (49.8)	9468 (42.8)		
Missing	1240 (5.6)	898 (4.1)	434 (2.0)	531 (2.4)			515 (2.3)	618 (2.8)	787 (3.6)	1212 (5.5)		
Alcohol‐drinking status, *n* (%)					<0.001	0.16					<0.001	0.08
Current	18442 (83.8)	19385 (88.1)	20388 (92.7)	19883 (90.4)			19904 (89.9)	19974 (90.2)	19785 (89.3)	18914 (85.4)		
Never	1786 (8.1)	1274 (5.8)	758 (3.4)	880 (4.0)			1099 (5.0)	1091 (4.9)	1191 (5.4)	1394 (6.3)		
Previous	1727 (7.8)	1299 (5.9)	846 (3.8)	1214 (5.5)			1114 (5.0)	1061 (4.8)	1143 (5.2)	1798 (8.1)		
Missing	45 (0.2)	41 (0.2)	7 (0.0)	22 (0.1)			29 (0.1)	20 (0.1)	26 (0.1)	39 (0.2)		
Smoking status, *n* (%)					<0.001	0.14					<0.001	0.13
Current	3344 (15.2)	2663 (12.1)	1968 (8.9)	2642 (12.0)			2213 (10.0)	2290 (10.3)	2658 (12.0)	3526 (15.9)		
Never	10639 (48.4)	10871 (49.4)	10823 (49.2)	9592 (43.6)			11558 (52.2)	10994 (49.6)	10413 (47.0)	9293 (42.0)		
Previous	7861 (35.7)	8349 (38.0)	9116 (41.4)	9669 (44.0)			8293 (37.4)	8750 (39.5)	8931 (40.3)	9207 (41.6)		
Missing	156 (0.7)	116 (0.5)	92 (0.4)	96 (0.4)			82 (0.4)	112 (0.5)	143 (0.6)	119 (0.5)		
Hearing impairment, *n* (%)					<0.001	0.04					<0.001	0.02
No	14943 (67.9)	14781 (67.2)	14665 (66.7)	14284 (64.9)			14803 (66.8)	14671 (66.2)	14839 (67.0)	14376 (64.9)		
Yes	6135 (27.9)	6284 (28.6)	6426 (29.2)	6819 (31.0)			6362 (28.7)	6415 (29.0)	6240 (28.2)	6671 (30.1)		
Missing	922 (4.2)	934 (4.2)	908 (4.1)	896 (4.1)			981 (4.4)	1060 (4.8)	1066 (4.8)	1098 (5.0)		
Obesity, *n* (%)					<0.001	0.08					<0.001	0.20
No	5321 (24.2)	5863 (26.7)	5544 (25.2)	4442 (20.2)			7207 (32.5)	5616 (25.4)	4778 (21.6)	3689 (16.7)		
Yes	16564 (75.3)	16066 (73.0)	16413 (74.6)	17480 (79.5)			14867 (67.1)	16453 (74.3)	17309 (78.2)	18344 (82.8)		
Missing	115 (0.5)	70 (0.3)	42 (0.2)	77 (0.4)			72 (0.3)	77 (0.3)	58 (0.3)	112 (0.5)		
Diabetes, *n* (%)					<0.001	0.08					<0.001	0.11
No	13711 (62.3)	14713 (66.9)	15145 (68.8)	14154 (64.3)			15350 (69.3)	14831 (67.0)	14386 (65.0)	13170 (59.5)		
Yes	7836 (35.6)	6893 (31.3)	6491 (29.5)	7386 (33.6)			6252 (28.2)	6778 (30.6)	7221 (32.6)	8370 (37.8)		
Missing	453 (2.1)	393 (1.8)	363 (1.7)	459 (2.1)			544 (2.5)	537 (2.4)	538 (2.4)	605 (2.7)		
Hypertension, *n* (%)					<0.001	0.05					<0.001	0.11
No	3170 (14.4)	3718 (16.9)	3862 (17.6)	3573 (16.2)			4516 (20.4)	3686 (16.6)	3229 (14.6)	2968 (13.4)		
Yes	18580 (84.5)	18031 (82.0)	17940 (81.5)	18208 (82.8)			17327 (78.2)	18206 (82.2)	18681 (84.4)	18970 (85.7)		
Missing	250 (1.1)	250 (1.1)	197 (0.9)	218 (1.0)			303 (1.4)	254 (1.1)	235 (1.1)	207 (0.9)		
Triglycerides, *n* (%)					<0.001	0.08					<0.001	0.18
Normal	8498 (38.6)	9354 (42.5)	9106 (41.4)	7829 (35.6)			10345 (46.7)	9187 (41.5)	8284 (37.4)	6988 (31.6)		
High	12496 (56.8)	11681 (53.1)	11900 (54.1)	13194 (60.0)			10636 (48.0)	11798 (53.3)	12769 (57.7)	14054 (63.5)		
Missing	1006 (4.6)	964 (4.4)	993 (4.5)	976 (4.4)			1165 (5.3)	1161 (5.2)	1092 (4.9)	1103 (5.0)		
Cholesterol, *n* (%)					<0.001	0.09					<0.001	0.20
Normal	10801 (49.1)	11845 (53.8)	12026 (54.7)	10811 (49.1)			12929 (58.4)	11930 (53.9)	11043 (49.9)	9598 (43.3)		
High	8931 (40.6)	7843 (35.7)	7626 (34.7)	8988 (40.9)			6578 (29.7)	7687 (34.7)	8761 (39.6)	10341 (46.7)		
Missing	2268 (10.3)	2311 (10.5)	2347 (10.7)	2200 (10.0)			2639 (11.9)	2529 (11.4)	2341 (10.6)	2206 (10.0)		

*Notes*: Q1–Q4 denote exposure quartiles (lowest to highest). *P*‐values are from *χ* ^2^ tests (categorical variables) or analysis of variance (ANOVA; continuous variables), comparing distributions across quartiles.

Abbreviations: *APOE*, apolipoprotein E; SD, standard deviation; SMD, standardized mean difference across quartiles.

### 3.2 Associations of computer use and TV viewing with dementia

Over a mean follow‐up of 12.2 ± 2.3 years, 2 282 participants developed dementia (945 AD, 540 VD, 75 FTD, 1 622 other). In fully adjusted Cox Model 3 (Table 2), each additional hour of computer use was linked to a 5% lower all‐cause dementia hazard (hazard ratio [HR] 0.95, 95% confidence interval [CI] 0.91–0.99; *p *= 0.010), yet a 6% higher hazard of AD (HR 1.06, 95% CI 1.04–1.09; *p *< 0.001) and other dementia (HR 1.06, 95% CI 1.02–1.10; *p* = 0.007), with no significant associations for VD (HR 0.95, 95% CI 0.89–1.01; *p *= 0.117) or FTD (HR 0.93, 95% CI 0.85–1.02; *p *= 0.106). Conversely, each extra hour of TV viewing conferred a 10% higher all‐cause hazard (HR 1.10, 95% CI 1.05–1.16; *p *< 0.001) and a 6% higher VD hazard (HR 1.06, 95% CI 1.03–1.10; *p *< 0.001), but was associated with a 7% lower AD hazard (HR 0.93, 95% CI 0.88–0.98; *p *= 0.005), and showed no significant links with other (HR 0.78, 95% CI 0.59–1.03; *p *= 0.079) or FTD (HR 1.05, 95% CI 0.91–1.22; *p *= 0.477).

**TABLE 2:** Multivariable Cox models of computer use and television viewing with all‐cause and subtype dementia outcomes.

Exposure (h/day)	Outcome	Model 1 (Age, sex, *APOE*)		Model 2 (+ SES and Lifestyle)		Model 3 (+ chronic conditions)	
		HR (95% CI)	*p*‐value	HR (95% CI)	*p*‐value	HR (95% CI)	*p*‐value
Computer use	All‐cause dementia	0.91 (0.88, 0.95)	< 0.001	0.95 (0.91, 0.98)	0.006	0.95 (0.91, 0.99)	0.010
	Alzheimer's disease	0.90 (0.85, 0.96)	0.001	1.07 (1.05, 1.10)	< 0.001	1.06 (1.04, 1.09)	< 0.001
	Vascular dementia	0.89 (0.82, 0.97)	0.006	0.96 (0.90, 1.02)	0.158	0.95 (0.89, 1.01)	0.117
	Frontotemporal dementia	0.71 (0.54, 0.93)	0.013	0.94 (0.87, 1.02)	0.147	0.93 (0.85, 1.02)	0.106
	Other dementia	0.89 (0.85, 0.93)	< 0.001	1.06 (1.02, 1.10)	0.005	1.06 (1.02, 1.10)	0.007
TV viewing	All‐cause dementia	1.12 (1.10, 1.15)	< 0.001	1.12 (1.07, 1.17)	< 0.001	1.10 (1.05, 1.16)	< 0.001
	Alzheimer's disease	1.10 (1.07, 1.14)	< 0.001	0.92 (0.88, 0.97)	0.001	0.93 (0.88, 0.98)	0.005
	Vascular dementia	1.18 (1.13, 1.22)	< 0.001	1.07 (1.04, 1.10)	< 0.001	1.06 (1.03, 1.10)	< 0.001
	Frontotemporal dementia	1.06 (0.94, 1.20)	0.344	1.05 (0.92, 1.20)	0.459	1.05 (0.91, 1.22)	0.477
	Other dementia	1.13 (1.10, 1.15)	< 0.001	0.73 (0.55, 0.97)	0.028	0.78 (0.59, 1.03)	0.079

*Notes*: Model 1: Adjusted for age, sex, and *APOE*‐ε4 genotype. Model 2: Model 1 + education level, family/friend visit frequency, ethnicity, Townsend deprivation index, physical activity, alcohol intake, and smoking status. Model 3: Model 2 + hearing impairment, obesity, diabetes, hypertension, high triglycerides, and total cholesterol.

Abbreviations: *APOE*, apolipoprotein E; CI, confidence interval; HR, hazard ratio.

### 3.3 Non‐linear dose–response

**FIGURE 1 alz71259-fig-0001:**
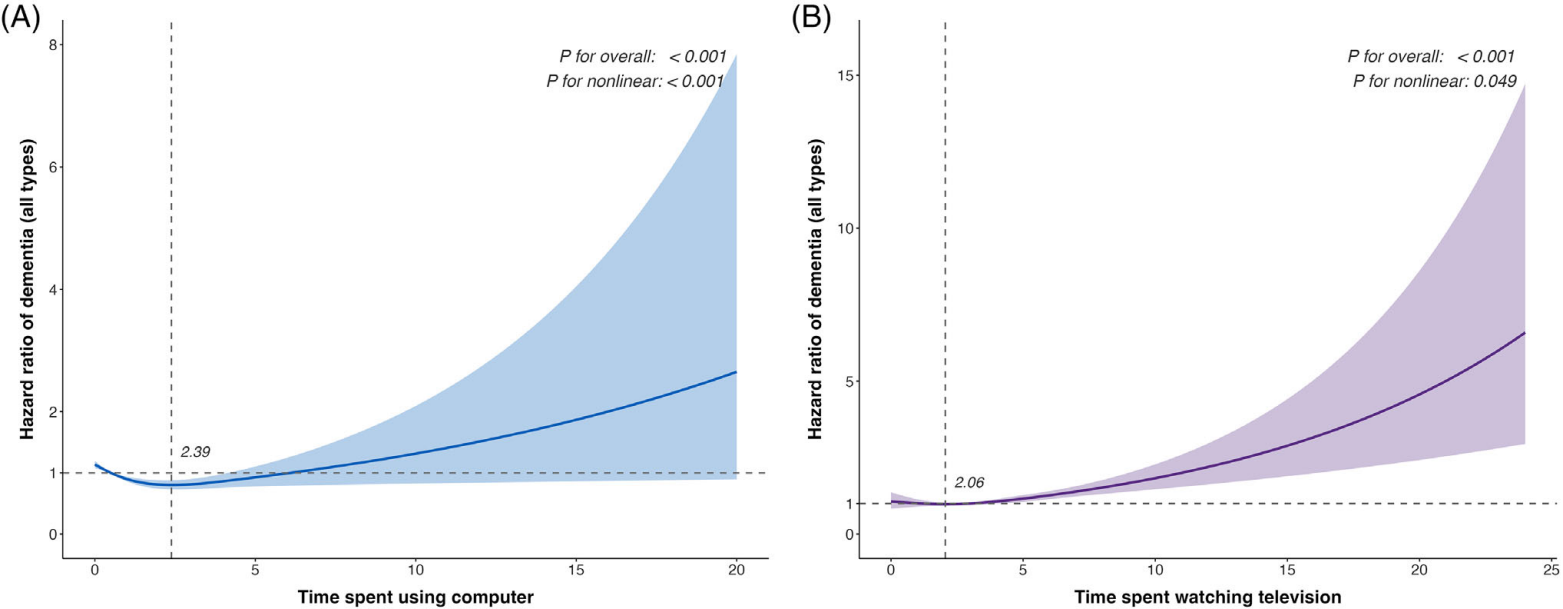
Multivariable‐adjusted hazard ratio curves for daily (A) computer use and (B) television viewing in relation to incident all‐cause dementia, modelled using four‐knot restricted cubic splines (RCS). Solid lines show the estimated hazard ratios (HRs) and shaded bands the 95% confidence intervals. The horizontal dashed line indicates HR = 1 (no effect), and the vertical dashed line indicates the RCS‐derived inflection point (2.39 h/day for computer use; 2.06 h/day for television [TV] viewing). Models were fully adjusted for age, sex, apolipoprotein E (*APOE*) genotype, education level, family/friend visit frequency, ethnicity, Townsend Deprivation Index, physical activity, alcohol intake, smoking status, hearing impairment, obesity, diabetes, hypertension, high triglycerides, and total cholesterol.

Restricted cubic splines (four knots) demonstrated significant non‐linear associations between computer use and all‐cause dementia (*p *< 0.001), AD (*p *< 0.001), VD (*p *= 0.002), and other dementia (*p *= 0.003), and for TV viewing with all‐cause dementia (*p *= 0.049) (Figure 1; Figure S2; Table 3). Piecewise Cox models identified inflection points at 2.39 h/day for all‐cause and VD and 2.19 h/day for AD. Below these thresholds, each additional hour of computer use reduced hazard (all‐cause HR 0.88, 95% CI 0.82–0.94; *p *< 0.001; AD HR 0.80, 95% CI 0.72–0.90; *p *< 0.001; VD HR 0.82, 95% CI 0.71–0.95; *p *= 0.007), whereas above them hazards increased (all‐cause HR 1.19, 95% CI 1.05–1.34; *p *= 0.005; AD HR 1.40, 95% CI 1.17–1.67; *p *< 0.001; VD HR 1.31, 95% CI 1.04–1.67; *p *= 0.024). No significant piecewise improvement was seen for computer use and other dementia (likelihood‐ratio *p* > 0.05). For TV viewing, the threshold was 2.06 h/day for all‐cause dementia; below it the association was non‐significant (HR 0.93, 95% CI 0.83–1.04; *p* = 0.207), whereas above it each additional hour conferred a 17% higher hazard (HR 1.17, 95% CI 1.03–1.32; *p* = 0.017).

**TABLE 3:** Piecewise Cox proportional‐hazards models for associations of computer use and TV viewing with all‐cause and subtype dementia outcomes, stratified by threshold.

Exposure (h/day)	Outcome	*p* for non‐linearity	Inflection (h/day)	HR below (95% CI)	*p* below	HR above (95% CI)	*p* above	LR *χ* ^2^	*p* for LR
Computer use	All‐cause dementia	< 0.001	2.39	0.88 (0.82, 0.94)	< 0.001	1.19 (1.05, 1.34)	0.005	7.27	0.007
	Alzheimer's disease	0.002	2.39	0.82 (0.71, 0.95)	0.007	1.31 (1.04, 1.67)	0.024	4.60	0.032
	Vascular dementia	< 0.001	2.19	0.80 (0.72, 0.90)	< 0.001	1.40 (1.17, 1.67)	< 0.001	12.00	0.001
	Other dementia	0.003	2.74	0.90 (0.83, 0.97)	0.007	1.10 (0.94, 1.29)	0.254	1.24	0.265
	Frontotemporal dementia	0.177	—	—	—	—	—	—	—
TV viewing	All‐cause dementia	0.049	2.06	0.93 (0.83, 1.04)	0.207	1.17 (1.03, 1.32)	0.017	5.45	0.020
	Alzheimer's disease	0.347	—	—	—	—	—	—	—
	Vascular dementia	0.238	—	—	—	—	—	—	—
	Other dementia	0.062	—	—	—	—	—	—	—
	Frontotemporal dementia	0.798	—	—	—	—	—	—	—

*Notes*: Inflection points (h/day) derive from four‐knot restricted cubic splines when non‐linearity *p* < 0.05; otherwise (*p* ≥ 0.05) piecewise estimates are omitted (—). “HR below” and “HR above” are hazard ratios per 1‐hour increase below/above the inflection, with two‐sided *p*‐values. “LR *χ* ^2^” and “*p* for LR” compare piecewise versus linear models (*p *< 0.05 indicates a better fit). Models were fully adjusted for age, sex, apolipoprotein E (*APOE*) genotype, education level, family/friend visit frequency, ethnicity, Townsend deprivation index, physical activity, alcohol intake, smoking status, hearing impairment, obesity, diabetes, hypertension, high triglycerides, and total cholesterol.

Abbreviations: CI, confidence interval; TV, television.

### 3.4 Interaction analysis of computer use and TV viewing

We observed no significant multiplicative interactions between computer use and TV viewing for all‐cause dementia (*p* for interaction = 0.371) or any dementia subtypes (all *p* for interaction > 0.05; Table S3). This indicates that the protective association of computer use and the adverse association of TV viewing operate independently.

### 3.5 Subgroup analysis

**FIGURE 2 alz71259-fig-0002:**
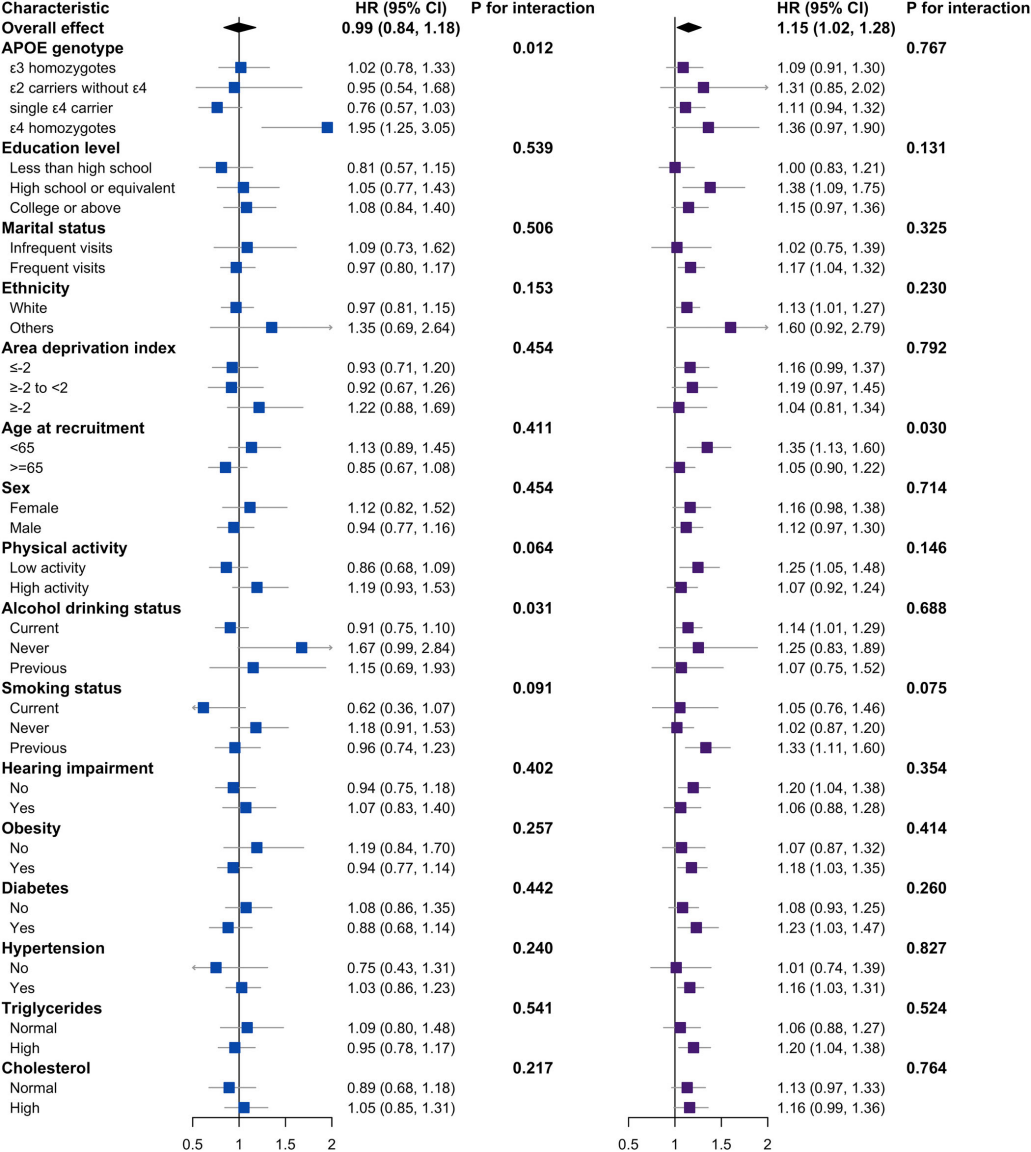
Subgroup analyses comparing high‐ versus low‐exposure thresholds and the hazard of incident all‐cause dementia. Panels display fully adjusted hazard ratios (HRs; squares) and 95% confidence intervals (horizontal lines) for computer use (left) and television (TV) viewing (right), comparing participants above versus below the spline‐derived inflection points (computer ≥ 2.39 h/day; TV ≥ 2.06 h/day). Diamonds indicate the overall HR in the full cohort. Models were fully adjusted for age, sex, apolipoprotein E (*APOE*) genotype, education level, family/friend visit frequency, ethnicity, Townsend deprivation index, physical activity, alcohol intake, smoking status, hearing impairment, obesity, diabetes, hypertension, high triglycerides, and total cholesterol.

For, all‐cause dementia (Figure 2), high computer use (≥2.39 h/day) interacted with *APOE*‐ε4 status (*p *= 0.012) and alcohol use (*p *= 0.031). In *APOE*‐ε4 homozygotes, high use nearly doubled risk (HR 1.95, 95% CI 1.25–3.05). Never‐ and former drinkers showed higher point estimates than current drinkers, but confidence intervals overlapped unity. No other interactions reached significance. High TV viewing (≥2.06 h/day) showed an age interaction (*p *= 0.030): participants < 65 years faced a 35% higher hazard (HR 1.35, 95% CI 1.13–1.60), with no association in those ≥65 years.

For AD (Figure S3), high computer use (2.19 h/day) interacted with *APOE*‐ε4 (*p *= 0.043) and physical activity (*p *= 0.029). *APOE*‐ε4 homozygotes had a two‐fold increased AD hazard (HR 2.16, 95% CI 1.19–3.91). High activity participants showed a non‐significant 36% risk increase (HR 1.36, 95% CI 0.94–1.97), whereas low‐activity individuals trended toward protection (HR 0.80, 95% CI 0.53–1.21). No other interactions were significant.

For VD (Figure S4), high computer use (≥2.39 h/day) interacted with physical activity (*p *= 0.025) and hearing impairment (*p *= 0.027). Among highly active participants, high use yielded a non‐significant 49% higher hazard (HR 1.49, 95% CI 0.94–2.36) versus a non‐significant 34% lower hazard in less active individuals (HR 0.66, 95% CI 0.39–1.11). Similarly, HRs were 1.34 (95% CI 0.85–2.11) in those with hearing impairment and 0.67 (95% CI 0.39–1.17) in those without—both nonsignificant.

### 3.6 Sensitivity analysis

Computer‐use associations were robust across sensitivity checks (multiple imputation, 2‐year landmark, Fine–Gray; Tables S4–S6): the inverse link with all‐cause dementia persisted (all *p* ≤ 0.017), as did its inverse association with other dementia (all *p* ≤ 0.010), whereas no associations emerged for AD or VD (all *p *> 0.05). FTD reached significance only in the imputation model (*p *= 0.019). When additionally adjusting for sleep duration and depressive symptoms (Table S7), the association between computer use and AD was attenuated to non‐significance (*p* = 0.133), while other associations remained unchanged. Similarly, TV‐viewing associations[Fig alz71259-fig-0001] were equally stable: elevated all‐cause dementia risk remained significant (all *p* < 0.001), as did positive links with AD (all *p* ≤ 0.044) and VD (all *p* ≤ 0.001), while no consistent effects appeared for other dementia or FTD (all *p *> 0.05). Notably, a significant positive association between TV viewing and “other dementia” emerged (*p* < 0.001), which was not significant in the primary models.

In the subgroup of socially active participants, TV viewing showed a linear association with dementia risk (*p* for non‐linear = 0.13), with each additional hour increasing the hazard by 9% (HR 1.09, 95% CI 1.07–1.11; *p* < 0.001). In contrast, computer use exhibited a strong non‐linear pattern (*p* for non‐linear < 0.001) despite a non‐significant linear trend (HR 0.98, *p* = 0.19). Piecewise analysis identified an inflection point for computer use at 0.63 h/day; usage below this threshold was protective (HR 0.53, 95% CI 0.46–0.60), whereas usage above it was associated with increased risk (HR 2.06, 95% CI 1.77–2.39).

## 4 DISCUSSION

In 89,671 socially isolated adults aged ≥55 years followed for 12.2 years, we identified novel, non‐linear, subtype‐specific associations between screen time and dementia. Computer use was associated with lower all‐cause dementia risk up to ∼2.36 h/day (12%–18% lower) but was associated with higher risk beyond this (19%–40% higher), while TV viewing was associated with higher all‐cause risk above ∼2.06 h/day (17% higher). Approximate linear associations were observed for subtypes: computer use was associated with a 6% higher risk of “other” dementia but not FTD, whereas TV viewing was associated with a 7% lower risk of AD and a 6% higher risk of VD. High computer use was associated with approximately twofold higher all‐cause and AD hazards in *APOE*‐ε4 homozygotes, and TV viewing ≥2.06 h/day was associated with 35% higher all‐cause risk in those < 65 years. These findings indicate apparent exposure optima that may inform risk stratification in similar populations; implications for behavioral modification (advocating interactive use within optimal limits, curbing passive viewing) remain hypothesis‐generating and require confirmation in interventional studies; future guidance may benefit from considering genetic, age, and lifestyle profiles.

Our results extend prior linear analyses of the inverse links between computer use and dementia risk,^8^, ^9^, ^10^, ^25^ revealing a J‐shaped association in socially isolated older adults. In this cohort, leisure computer use up to ∼2.4 h/day was association with lower all‐cause, VD, and AD risk by 12%–18%, but beyond this threshold was associated with 19%–40% higher incidence. Mechanistically, the observed inflection may reflect the point at which cognitive gains from interactive tasks are outweighed by the physiological and psychosocial costs of prolonged sedentary screen time. Below ∼2.4 h/day, activities such as reading, puzzles, video calls, and online learning appear to recruit prefrontal and hippocampal circuits, support synaptic plasticity, increase brain‐derived neurotrophic factor, and contribute to cognitive reserve,^26^, ^27^, ^28^ even after adjusting for physical inactivity. Reinforcing this, the protective association with AD became non‐significant after adjusting for sleep and depressive symptoms, suggesting the benefits of active engagement are largely mediated by improved sleep and mental health. Beyond this threshold, any incremental advantage seems to level off as mental fatigue, blue‐light–related sleep disruption, impaired cerebral perfusion and glymphatic clearance, and isolation‐related stress accumulate,^29^, ^30^, ^31^ yielding the J‐shaped dose‐response. In contrast, younger, socially engaged United Kingdom and United States cohorts report inflection points near 2 h/day,^12^, ^32^ which may reflect higher baseline cognitive reserve and more leisure options that saturate enrichment and accumulate harm sooner. Notably, we observed no association between computer use and FTD, consistent with FTD's earlier onset, less influenced by late‐life lifestyle factors, and a stronger genetic contribution than AD or VD.^33^ This null finding could also reflect outcome misclassification given UK Biobank's algorithmic ascertainment of FTD (non‐specific International Classification of Diseases [ICD] codes; small case numbers). Accordingly, ≤2.4 h/day of cognitively engaging computer use can serve as a pragmatic benchmark for risk tools, with flags for > 2.4 h/day or for prolonged continuous bouts (> 60 min). As a candidate intervention (to be evaluated in trials), a digital‐literacy program that emphasizes active tasks (e.g., video calls, puzzles, online learning), pacing (≤60‐minute bouts with 5‐ to 10‐minute breaks), and weekly self‐monitoring should be tested for feasibility and effectiveness.[Fig alz71259-fig-0002]

In contrast, even after adjusting for overall inactivity, TV viewing above ∼2 h/day independently raises dementia risk, likely via several pathways. Passive viewing fails to engage frontal and temporal networks, displacing protective cognitive and social activities. Neuroimaging links prolonged TV exposure to declines in grey‐matter microstructure—reduced neurite density in temporal and frontal regions critical for memory and executive function—consistent with “disuse atrophy” of cognitive networks.^7^, ^34^ Prolonged sitting impairs endothelial function and insulin sensitivity, accelerating microvascular brain damage.^35^ Evening screen exposure disrupts sleep and glymphatic clearance of neurotoxins,^36^ while passive TV lacks social reciprocity, even in online‐formats, sustaining isolation and neuroinflammation. However, an earlier study linked > 3.5 h/day of TV viewing to 6‐year verbal‐memory decline;^34^ its higher threshold likely reflects a younger, more active cohort, shorter follow‐up, and fewer covariate adjustments. Notably, our inverse TV–AD association, though significant, was attenuated in competing‐risk models and was not robust in sensitivity analyses (e.g., 2‐year landmarking). This pattern is more consistent with non‐causal explanations, including reverse causation in prodromal AD (apathy and attentional and comprehension deficits altering TV behavior) and diagnostic substitution (misclassification between AD and vascular dementia in routine records). Notably, additional adjustment for sleep and depressive symptoms unmasked a significant association between TV viewing and “other dementia”, suggesting psychological factors previously suppressed this specific risk. Additionally, our restriction to individuals reporting no leisure or social activities may induce collider bias: because both pre‐clinical AD traits and television viewing are associated with social inactivity, conditioning on inactivity can produce artifactual inverse associations. Therefore, we interpret the apparent protective TV–AD association as non‐causal and likely spurious. For risk stratification, TV viewing ≥2.0 h/day, particularly in the evening, can trigger targeted counselling; candidate interventions to evaluate include daily caps (e.g., ≤120 min/day), hourly interruption prompts, replacing ≥30–60 min/day with socially or cognitively engaging alternatives, and sleep‐hygiene guidance to limit late‐evening viewing.

Subgroup analyses highlight the vulnerability of ε4 homozygotes (almost double all‐cause and AD risk with higher computer use) and adults < 65 years (35% higher all‐cause risk with high TV viewing). *APOE*‐ε4 homozygotes, whose cognitive reserve is already compromised by early amyloid deposition,^37^ were particularly susceptible to prolonged interactive screen time, likely overwhelming synaptic networks under metabolic stress and doubling dementia risk. Neuroplasticity and brain resilience decline with age,^38^ making midlife a critical window when lifestyle exerts outsized effects on later cognition. During this period, excessive TV viewing is linked to insulin resistance, endothelial dysfunction, and accelerated grey‐matter loss in frontal and temporal regions—early pathological changes preceding dementia by decades^39^, ^40^ —thereby elevating all‐cause dementia risk in adults < 65. Survivor bias may also attenuate associations in those ≥65. Although interactions by factors such as alcohol use, physical activity, and hearing loss were significant, within‐stratum effects were null and imprecise, likely due to small subgroup sizes. Future studies should use larger pooled cohorts to refine subgroup risk estimates. For risk stratification, flag *APOE*‐ε4 homozygotes reporting interactive computer use > ∼2.4 h/day and adults < 65 years reporting television viewing ≥2.0 h/day. These groups should also be prioritized for testing candidate interventions, with effects confirmed in interventional studies.

TV viewing followed a linear trajectory in the socially active group, contrasting with the threshold effect in the inactive group. This suggests that for isolated individuals, TV viewing becomes detrimental only beyond a specific tipping point, likely because moderate viewing may buffer loneliness. Conversely, the threshold for harm from computer use was substantially lower in active adults (0.63 vs. 2.39 h/day). This supports the time displacement hypothesis: for socially active individuals, even moderate screen use likely competes with essential restorative behaviors (e.g., sleep, physical activity), whereas for inactive adults, computer use provides otherwise lacking cognitive stimulation. Consequently, screen guidelines should be tailored to social context; strict limitations may benefit socially active individuals but could deprive isolated older adults of a critical cognitive resource.

### 4.1 Limitations

Several limitations warrant consideration. First, as an observational cohort study, causal relationships cannot be inferred; observed associations may reflect residual or unmeasured confounding and reverse causation. Second, a single baseline self‐report likely introduced measurement error, missed secular shifts in digital‐media use, and lacked content/context; session fragmentation, breaks, concurrent media, timing (e.g., evening vs daytime), and device type were unavailable. Objective device logs with repeated measures would better capture dynamic exposure. Third, in UK Biobank, “computer use” aggregated non‐work activities, conflating cognitively engaging/socially interactive behaviors (e.g., video calls, online learning, problem‐solving games) with passive use (e.g., autoplay browsing), limiting activity‐specific associations with dementia risk. Fourth, the analytic sample was drawn largely from socially isolated, predominantly White adults living in a high‐income country, which may limit generalizability to lower‐resource settings, ethnically diverse groups, and distinct cultural contexts. Fifth, secular increases in screen availability and earlier dementia detection during the 12‐year follow‐up could bias associations, emphasizing the need for replication in cohorts with time‐varying exposures and outcomes. Future work should incorporate advanced neuroimaging, particularly resting‐state fMRI to characterize cognitive‐network activity, and fluid biomarkers, including plasma phosphorylated tau217 (p‐tau217) and other blood‐based Alzheimer's markers, test interventions that reduce passive TV viewing or promote moderate computer use with activity breaks, and examine whether similar non‐linear, genotype‐ and biomarker‐modified patterns emerge in broader or more socially engaged populations.

## 5 CONCLUSIONS

In this large cohort of socially isolated adults aged ≥55 years, interactive computer use was associated with lower dementia incidence up to a moderate daily duration (∼2.4 h/day) but was associated with higher incidence beyond, whereas passive TV viewing was associated with higher incidence only above ∼2 h/day. These non‐linear, subtype‐specific associations, and their modification by *APOE*‐ε4 status and age, may inform risk stratification and hypothesis generation for trials. These observational findings indicate apparent exposure optima that may aid risk stratification in similar populations as candidate targets for counselling and monitoring, for example, flagging computer use > ∼2.4 h/day and television viewing ≥2.0 h/day, particularly in *APOE*‐ε4 carriers and younger adults. They may also inform candidate intervention strategies, such as provisional screen‐use ranges that promote interactive computer use within putative optimal doses and limit prolonged passive viewing, but effects remain unproven. Whether modifying screen use within these ranges alters dementia risk is uncertain; confirmation with repeated, content‐resolved exposure measures, more diverse cohorts, and interventional studies (ideally incorporating biomarkers and neuroimaging) is needed to assess causality and refine personalized, risk‐balanced guidance.

## AUTHOR CONTRIBUTIONS

JX Li and JY Li designed the study. JY Li conducted the data analysis and drafted the manuscript. H. Xue assisted with variable selection. Y. Leng, Q.M. Samus, M. Nowrangi, S.L. Szanton, Q. Xue, and JX. Li contributed to critical revision of the manuscript for important intellectual content. All authors approved the final version and agree to be accountable for all aspects of the work.

## CONFLICT OF INTEREST STATEMENT

The authors (JY. Li, H. Xue, Y. Leng, Q.M. Samus, M. Nowrangi, S.L. Szanton, Q. Xue, and JX. Li) declare that they have no conflicts of interest relevant to this work.

## ETHICS STATEMENT

UK Biobank has obtained ethical approval from the North West Multi‐center Research Ethics Committee (REC reference 11/NW/0382). The present analysis was conducted under UK Biobank application number [755949], in accordance with the Declaration of Helsinki and applicable regulations.

## CONSENT STATEMENT

All participants provided written informed consent at enrolment.

## Supporting information



Supporting Information

Supporting Information

## Data Availability

UK Biobank data are available upon application to the UK Biobank Access Management System (www.ukbiobank.ac.uk/enable‐your‐research) under the terms of the Material Transfer Agreement; the dataset used in this study cannot be shared publicly by the authors.
