# Supplementary material for: Television and computer use and dementia risk in older adults with limited leisure or social activities: A prospective cohort study
Source: Alzheimers Dement. 2026 Mar 8;22(3):e71259. doi: 10.1002/alz.71259 (PMC12967449; doi:10.1002/alz.71259)
Supplement: Supplementary file 1 — Supporting Information [file ALZ-22-e71259-s002.docx]

**Supplementary materials**

| **Tables or figures** | **Page number** |
| --- | --- |
| **Supplementary Table 1**. International Classification of Diseases (ICD)-10 and ICD-9 code definitions for incident all-cause dementia and its subtypes. | 1 |
| **Supplementary Table 2.** Missing data rates and covariate imputation methods using multiple imputation by chained equations (MICE). | 2 |
| **Supplementary Table 3.** Interaction analysis of computer use and TV viewing on dementia risk. | 3 |
| **Supplementary Table 4.** Sensitivity analysis results after multiple imputation of missing covariates. | 4 |
| **Supplementary Table *5*.** Sensitivity analysis results from 2-year landmark (left-truncation) models to mitigate reverse causality. | 5 |
| **Supplementary Table 6.** Sensitivity analysis results from Fine–Gray competing-risk models accounting for mortality. | 6 |
| **Supplementary Table 7.** Sensitivity analysis results after additional adjustment for sleep duration and depressive symptoms. | 7 |
| **Supplementary Figure 1.** Flowchart of participant selection from the UK Biobank to the analytic cohort (n = 89 671). | 8 |
| **Supplementary Figure 2**. Multivariable-adjusted hazard ratio curves for daily computer use and television viewing in relation to incident dementia subtypes, modelled with four-knot restricted cubic splines. | 9 |
| **Supplementary Figure 3.** Subgroup analyses comparing high‐ versus low‐exposure thresholds and the hazard of incident Alzheimer’s dementia. | 10 |
| **Supplementary Figure 4.** Subgroup analyses comparing high‐ versus low‐exposure thresholds and the hazard of incident vascular dementia. | 11 |

**Supplementary Table 1**. International Classification of Diseases (ICD)-10 and ICD-9 code definitions for incident all-cause dementia and its subtypes.

| **Dementia subtype** | **ICD-10 codes** | **ICD-9 codes** |
| --- | --- | --- |
| Alzheimer disease | F00.0, F00.1, F00.2, F00.9, G30.0, G30.1, G30.8, G30.9 | 331 |
| Vascular dementia | F01.0, F01.1, F01.2, F01.3, F01.8, F01.9, I67.3 | 290.4 |
| Frontotemporal dementia | F02.0, G31.0 | 331.1 |
| Other dementia | A81.0, F02.1, F02.2, F02.3, F02.4, F02.8, F03, F05.1, F10.6, G31.1, G31.8 | 290.2, 290.3, 291.2, 294.1, 331.2, 331.5 |

***Note***: International Classification of Diseases: ICD.

**Supplementary Table 2.** Missing data rates and covariate imputation methods using multiple imputation by chained equations (MICE).

| **Covariates** | **Missing rate** | **Imputation method** |
| --- | --- | --- |
| APOE genotype | 3.0% | polyreg |
| Education level | 2.0% | polyreg |
| Social visit frequency | 1.4% | logreg |
| Ethnicity | 0.4% | logreg |
| Area deprivation index | 0.1% | polyreg |
| Physical activity | 3.9% | logreg |
| Alcohol drinking status | 0.1% | polyreg |
| Smoking status | 0.6% | polyreg |
| Hearing impairment | 4.8% | logreg |
| Obesity | 0.4% | logreg |
| Diabetes | 2.5% | logreg |
| Hypertension | 1.1% | logreg |
| Triglycerides | 5.1% | logreg |
| Cholesterol | 11.0% | logreg |

***Note:*** APOE, apolipoprotein E; polyreg, polytomous regression; logreg, logistic regression.

**Supplementary Table 3.** Interaction analysis of computer use and TV viewing on dementia risk.

| **Outcome** | **Interaction term** | **HR (95% CI)** | **P for interaction** |
| --- | --- | --- | --- |
| All-cause dementia | Computer × TV | 1.01 (0.99-1.02) | 0.371 |
| Alzheimer’s disease | Computer × TV | 1.01 (0.98-1.04) | 0.631 |
| Vascular dementia | Computer × TV | 0.99 (0.95-1.03) | 0.540 |
| Frontotemporal dementia | Computer × TV | 1.03 (0.94-1.14) | 0.543 |
| Other dementia | Computer × TV | 1.01 (0.99-1.03) | 0.262 |

***Note***: Adjusted for age, sex, apolipoprotein E ε4 (APOE-ε4) genotype, education level, family/friend visit frequency, ethnicity, Townsend deprivation index, physical activity, alcohol intake, smoking status, hearing impairment, obesity (body mass index ≥ 30 kg/m²), diabetes, hypertension, high triglycerides, and total cholesterol.

HR, hazard ratio; CI, confidence interval.

**Supplementary Table 4.** Sensitivity analysis results after multiple imputation of missing covariates.

| **Outcome** | **Exposure (h/day)** | **Model 1** | | **Model 2** | | **Model 3** | |
| --- | --- | --- | --- | --- | --- | --- | --- |
|  |  | ***HR* (95% *CI*)** | ***p*-value** | ***HR* (95% *CI*)** | ***p*-value** | ***HR* (95% CI)** | ***p*-value** |
| All-cause dementia | Computer | 0.91 (0.88–0.95) | <0.001 | 0.95 (0.91–0.98) | 0.003 | 0.95 (0.91–0.98) | 0.002 |
| Alzheimer’s disease (AD) | Computer | 0.90 (0.85–0.96) | 0.001 | 0.95 (0.90–1.01) | 0.080 | 0.95 (0.90–1.01) | 0.086 |
| Vascular dementia (VD) | Computer | 0.90 (0.83–0.97) | 0.008 | 0.94 (0.88–1.02) | 0.14 | 0.94 (0.87–1.01) | 0.085 |
| Frontotemporal dementia | Computer | 0.70 (0.53–0.92) | 0.009 | 0.72 (0.55–0.94) | 0.016 | 0.72 (0.55–0.95) | 0.019 |
| Other dementia | Computer | 0.89 (0.85–0.93) | <0.001 | 0.93 (0.89–0.97) | 0.001 | 0.93 (0.89–0.97) | 0.001 |
| All-cause dementia | TV viewing | 1.12 (1.10–1.14) | <0.001 | 1.08 (1.05–1.10) | <0.001 | 1.07 (1.05–1.10) | <0.001 |
| Alzheimer’s disease (AD) | TV viewing | 1.10 (1.06–1.13) | <0.001 | 1.05 (1.02–1.09) | 0.005 | 1.05 (1.02–1.09) | 0.004 |
| Vascular dementia (VD) | TV viewing | 1.19 (1.15–1.23) | <0.001 | 1.15 (1.10–1.19) | <0.001 | 1.13 (1.09–1.18) | <0.001 |
| Frontotemporal dementia | TV viewing | 1.05 (0.93–1.19) | 0.403 | 1.02 (0.90–1.16) | 0.760 | 1.04 (0.91–1.18) | 0.569 |
| Other dementia | TV viewing | 1.12 (1.09–1.15) | <0.001 | 1.08 (1.05–1.10) | <0.001 | 1.08 (1.05–1.10) | <0.001 |

***Note***: we generated five imputed datasets via multiple imputation by chained equations and combined estimates using Rubin’s rules for this sensitivity analyses; Model 1: Adjusted for age, sex, and apolipoprotein E ε4 (APOE-ε4) genotype.

Model 2: Model 1 + education level, family/friend visit frequency, ethnicity, Townsend deprivation index, physical activity, alcohol intake, and smoking status.

Model 3: Model 2 + hearing impairment, obesity (body mass index ≥ 30 kg/m²), diabetes, hypertension, high triglycerides, and total cholesterol.

HR, hazard ratio; CI, confidence interval.

**Supplementary Table 5.** Sensitivity analysis results from 2-year landmark (left-truncation) models to mitigate reverse causality.

| **Outcome** | **Exposure (h/day)** | **Model 1** | | **Model 2** | | **Model 3** | |
| --- | --- | --- | --- | --- | --- | --- | --- |
|  |  | ***HR* (95% *CI*)** | ***p*-value** | ***HR* (95% *CI*)** | ***p*-value** | ***HR* (95% *CI*)** | ***p*-value** |
| All-cause dementia | Computer | 0.92 (0.88–0.95) | <0.001 | 0.95 (0.91–0.99) | 0.010 | 0.95 (0.91–0.99) | 0.017 |
| Alzheimer’s disease | Computer | 0.90 (0.85–0.96) | 0.001 | 0.95 (0.90–1.01) | 0.126 | 0.94 (0.88–1.01) | 0.100 |
| Vascular dementia | Computer | 0.90 (0.83–0.98) | 0.010 | 0.95 (0.87–1.03) | 0.188 | 0.94 (0.86–1.02) | 0.130 |
| Frontotemporal dementia | Computer | 0.71 (0.54–0.93) | 0.012 | 0.73 (0.55–0.97) | 0.027 | 0.77 (0.58–1.03) | 0.076 |
| Other dementia | Computer | 0.90 (0.86–0.94) | <0.001 | 0.93 (0.89–0.98) | 0.003 | 0.94 (0.89–0.99) | 0.010 |
| All-cause dementia | TV viewing | 1.12 (1.10–1.15) | <0.001 | 1.07 (1.05–1.10) | <0.001 | 1.06 (1.04–1.09) | <0.001 |
| Alzheimer’s disease | TV viewing | 1.11 (1.07–1.14) | <0.001 | 1.06 (1.02–1.10) | 0.005 | 1.06 (1.02–1.10) | 0.007 |
| Vascular dementia | TV viewing | 1.18 (1.14–1.22) | <0.001 | 1.12 (1.07–1.17) | <0.001 | 1.10 (1.05–1.15) | <0.001 |
| Frontotemporal dementia | TV viewing | 1.06 (0.94–1.20) | 0.329 | 1.05 (0.92–1.20) | 0.458 | 1.05 (0.91–1.22) | 0.486 |
| Other dementia | TV viewing | 1.13 (1.10–1.15) | <0.001 | 1.07 (1.04–1.10) | <0.001 | 1.06 (1.03–1.10) | <0.001 |

***Note***: Model 1: Adjusted for age, sex, and apolipoprotein E ε4 (APOE-ε4) genotype.

Model 2: Model 1 + education level, family/friend visit frequency, ethnicity, Townsend deprivation index, physical activity, alcohol intake, and smoking status.

Model 3: Model 2 + hearing impairment, obesity (body mass index ≥ 30 kg/m²), diabetes, hypertension, high triglycerides, and total cholesterol.

HR, hazard ratio; CI, confidence interval.

**Supplementary Table 6.** Sensitivity analysis results from Fine–Gray competing-risk models accounting for mortality.

| **Outcome** | **Exposure (h/day)** | **Model 1** | | **Model 2** | | **Model 3** | |
| --- | --- | --- | --- | --- | --- | --- | --- |
|  |  | ***HR* (95% *CI*)** | ***p*-value** | ***HR* (95% *CI*)** | ***p*-value** | ***HR* (95% *CI*)** | ***p*-value** |
| All-cause dementia | Computer | 0.91 (0.88–0.95) | <0.001 | 0.95 (0.91–0.99) | 0.010 | 0.95 (0.90–0.99) | 0.014 |
| Alzheimer’s disease | Computer | 0.91 (0.84–0.98) | 0.012 | 0.96 (0.89–1.03) | 0.220 | 0.95 (0.87–1.02) | 0.160 |
| Vascular dementia | Computer | 0.90 (0.82–0.98) | 0.020 | 0.94 (0.86–1.03) | 0.180 | 0.93 (0.84–1.02) | 0.130 |
| Frontotemporal dementia | Computer | 0.72 (0.53–0.97) | 0.032 | 0.74 (NA–NA) | NA | 0.78 (0.59–1.04) | 0.093 |
| Other dementia | Computer | 0.89 (0.85–0.94) | <0.001 | 0.92 (0.88–0.97) | 0.003 | 0.93 (0.88–0.98) | 0.006 |
| All-cause dementia | Television | 1.11 (1.08–1.13) | <0.001 | 1.06 (1.04–1.09) | <0.001 | 1.05 (1.02–1.08) | <0.001 |
| Alzheimer’s disease | Television | 1.09 (1.05–1.12) | <0.001 | 1.04 (1.00–1.09) | 0.035 | 1.05 (1.00–1.09) | 0.044 |
| Vascular dementia | Television | 1.16 (1.12–1.21) | <0.001 | 1.11 (1.06–1.16) | <0.001 | 1.09 (1.03–1.15) | 0.001 |
| Frontotemporal dementia | Television | 1.05 (0.93–1.18) | 0.440 | 1.04 (0.92–1.19) | 0.510 | 1.05 (0.90–1.21) | 0.540 |
| Other dementia | Television | 1.11 (1.08–1.14) | <0.001 | 1.06 (1.03–1.09) | <0.001 | 1.05 (1.02–1.09) | 0.002 |

***Note***: Model 1: Adjusted for age, sex, and apolipoprotein E ε4 (APOE-ε4) genotype.

Model 2: Model 1 + education level, family/friend visit frequency, ethnicity, Townsend deprivation index, physical activity, alcohol intake, and smoking status.

Model 3: Model 2 + hearing impairment, obesity (body mass index ≥ 30 kg/m²), diabetes, hypertension, high triglycerides, and total cholesterol.

HR, hazard ratio; CI, confidence interval.

**Supplementary Table 7.** Sensitivity analysis results after additional adjustment for sleep duration and depressive symptoms.

| **Outcome** | **Exposure (h/day)** | ***HR* (95% *CI*)** | ***p*-value** |
| --- | --- | --- | --- |
| All-cause dementia | Computer | 0.95 (0.91, 0.99) | 0.015 |
| Alzheimer’s disease | Computer | 0.95 (0.89, 1.02) | 0.133 |
| Vascular dementia | Computer | 0.93 (0.85, 1.02) | 0.105 |
| Frontotemporal dementia | Computer | 0.78 (0.59, 1.03) | 0.078 |
| Other dementia | Computer | 0.93 (0.89, 0.98) | 0.007 |
| All-cause dementia | Television | 1.06 (1.03, 1.09) | < 0.001 |
| Alzheimer’s disease | Television | 1.05 (1.01, 1.10) | 0.016 |
| Vascular dementia | Television | 1.10 (1.04, 1.15) | < 0.001 |
| Frontotemporal dementia | Television | 1.06 (0.91, 1.22) | 0.450 |
| Other dementia | Television | 1.06 (1.03, 1.09) | < 0.001 |

***Note***: Adjusted for age, sex, apolipoprotein E ε4 (APOE-ε4) genotype, education level, family/friend visit frequency, ethnicity, Townsend deprivation index, physical activity, alcohol intake, smoking status, hearing impairment, obesity (body mass index ≥ 30 kg/m²), diabetes, hypertension, high triglycerides, total cholesterol, sleep duration (normal [7–9 h] vs. non-normal [<7 h or >9 h]), and depressive symptoms (dichotomized according to the Patient Health Questionnaire-2 [PHQ-2] standard cut-off).

HR, hazard ratio; CI, confidence interval.

**Supplementary Figure 1.** Flowchart of participant selection from the UK Biobank to the analytic cohort (n = 89 671).


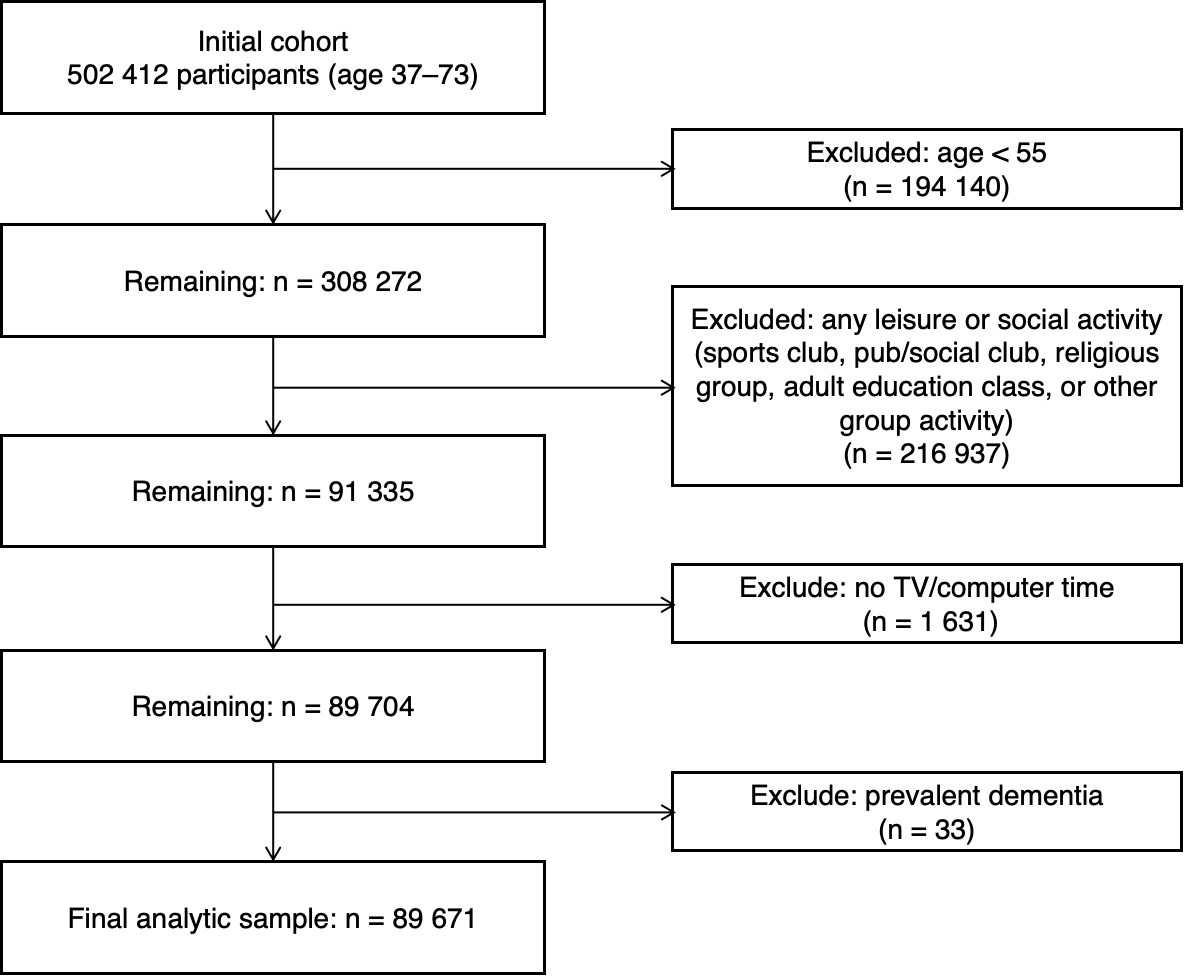


***Note***: TV, television.

**Supplementary Figure 2**. Multivariable-adjusted hazard ratio curves for daily computer use and television viewing in relation to incident dementia subtypes, modelled with four-knot restricted cubic splines.


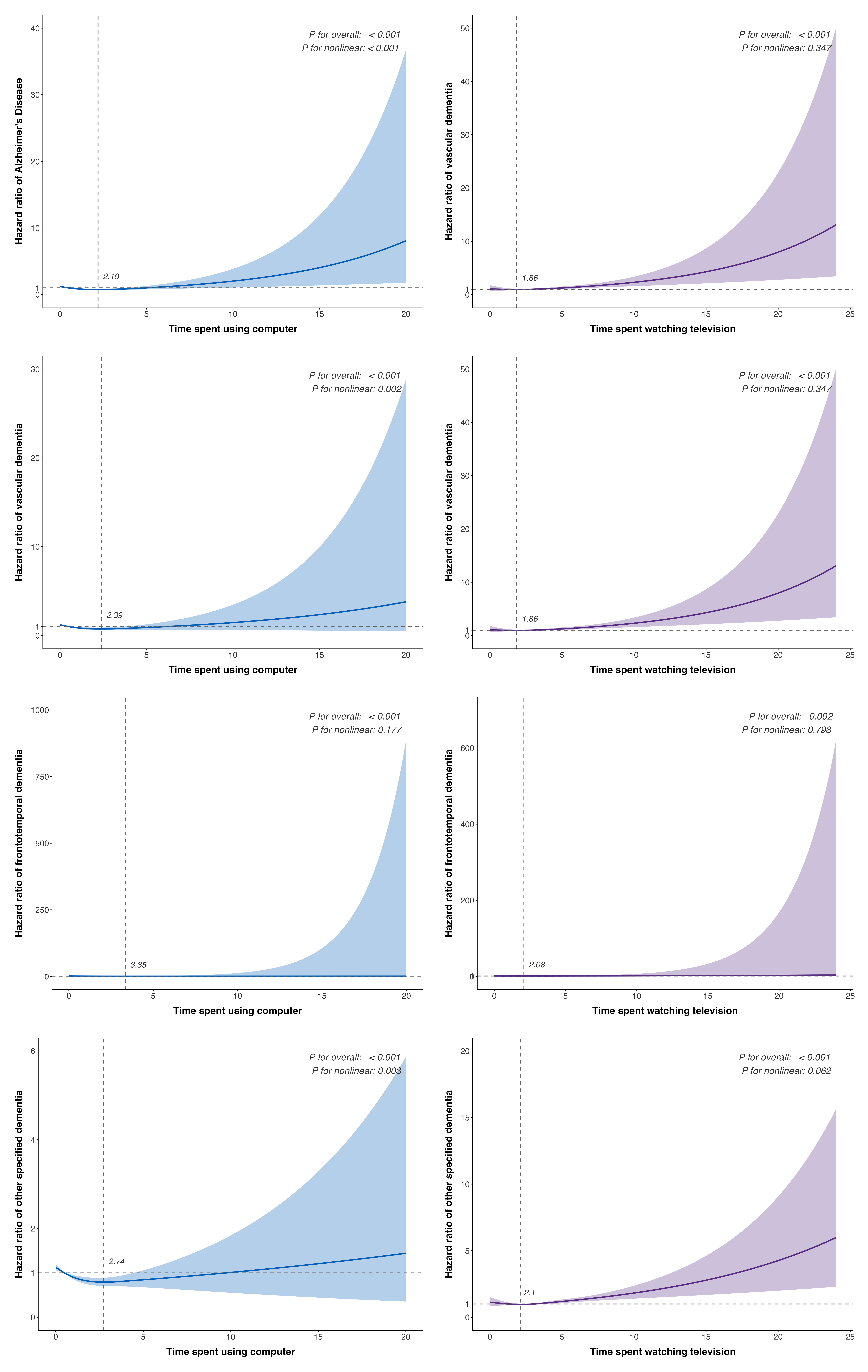


***Note***: Solid lines show the estimated hazard ratios (HRs) and shaded bands the 95% confidence intervals. The horizontal dashed line indicates HR = 1 (no effect), and the vertical dashed line indicates the restricted cubic spline–derived inflection point. Models were fully adjusted for age, sex, apolipoprotein E (APOE) genotype, education level, family/friend visit frequency, ethnicity, Townsend deprivation index, physical activity, alcohol intake, smoking status, hearing impairment, obesity, diabetes, hypertension, high triglycerides, and total cholesterol.

**Supplementary Figure 3.** Subgroup analyses comparing high‐ versus low‐exposure thresholds and the hazard of incident Alzheimer’s dementia.


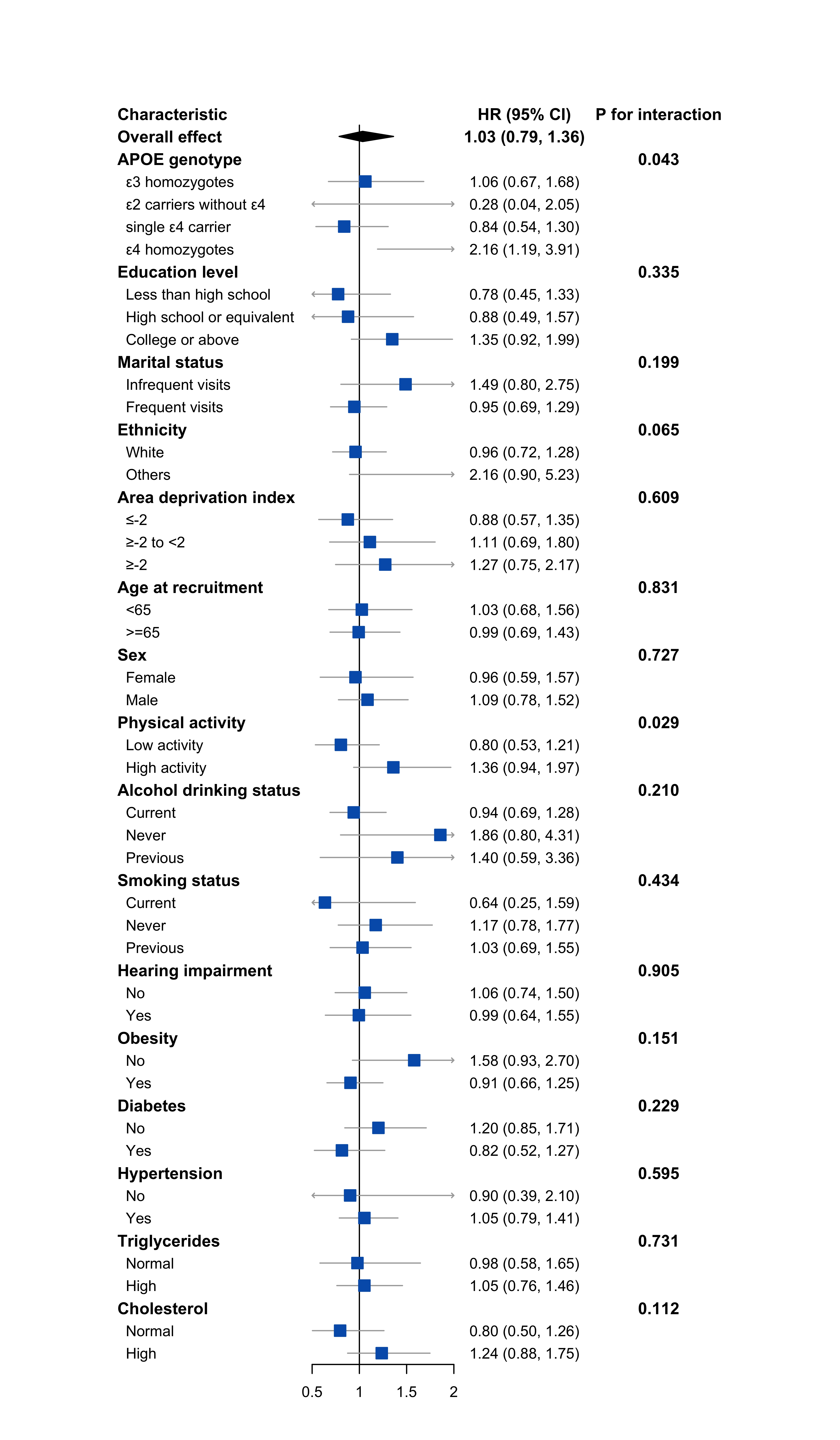


***Note***: Panels display fully adjusted hazard ratios (HRs; squares) and 95% confidence intervals (horizontal lines) for computer use, comparing participants above versus below the spline-derived inflection points (computer ≥ 2.39 h/day). Diamonds indicate the overall HR in the full cohort. Models were fully adjusted for age, sex, apolipoprotein E (APOE) genotype, education level, family/friend visit frequency, ethnicity, Townsend deprivation index, physical activity, alcohol intake, smoking status, hearing impairment, obesity, diabetes, hypertension, high triglycerides, and total cholesterol.

**Supplementary Figure 4.** Subgroup analyses comparing high‐ versus low‐exposure thresholds and the hazard of incident vascular dementia.


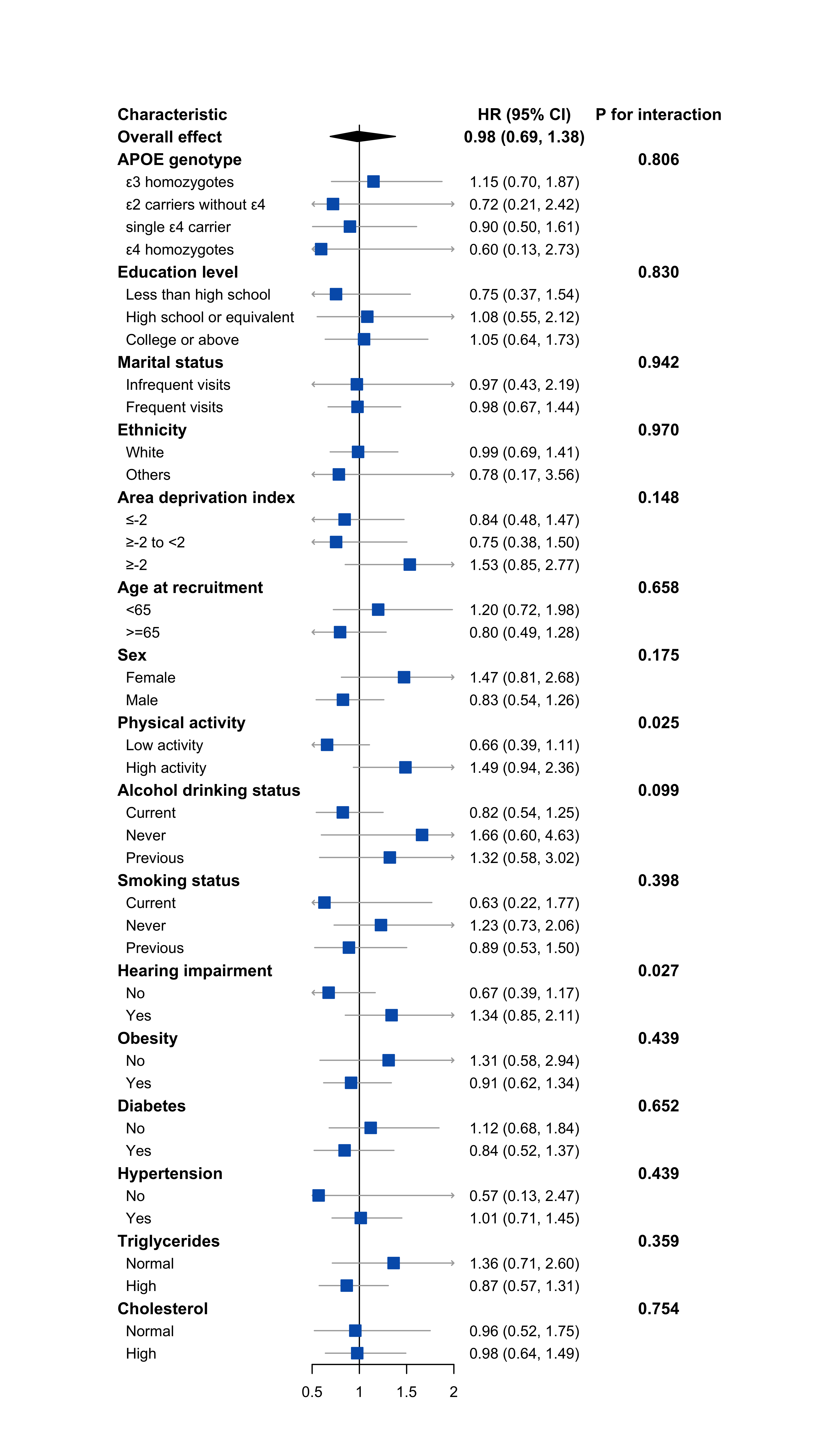


***Note***: Panels display fully adjusted hazard ratios (HRs; squares) and 95% confidence intervals (horizontal lines) for computer use, comparing participants above versus below the spline-derived inflection points (computer ≥ 2.19 h/day). Diamonds indicate the overall HR in the full cohort. Models were fully adjusted for age, sex, apolipoprotein E (APOE) genotype, education level, family/friend visit frequency, ethnicity, Townsend deprivation index, physical activity, alcohol intake, smoking status, hearing impairment, obesity, diabetes, hypertension, high triglycerides, and total cholesterol.
